# Adult Smoking Cessation — United States, 2022

**DOI:** 10.15585/mmwr.mm7329a1

**Published:** 2024-07-25

**Authors:** Brenna VanFrank, Ann Malarcher, Monica E. Cornelius, Anna Schecter, Ahmed Jamal, Michael Tynan

**Affiliations:** ^1^Office on Smoking and Health, National Center for Chronic Disease Prevention and Health Promotion, CDC; ^2^Katmai Government Services, Anchorage, Alaska.

**Summary**

**What is already known about this topic?**

Evidence-based treatment and clinician intervention increase successful smoking cessation.

**What is added by this report?**

In 2022, the majority of the 28.8 million U.S. adults who smoked cigarettes wanted to quit, approximately one half tried to quit, but fewer than 10% were successful. Fewer than 40% of adults who smoked used treatment (counseling or medication) when trying to quit; one half received clinician advice or assistance to quit. Compared with adults who smoked nonmenthol cigarettes, those who smoked menthol cigarettes had similarly low quit success despite a higher quit attempt prevalence, potentially related to their lower treatment use.

**What are the implications for public health practice?**

Increasing access to and use of smoking cessation services and incorporating equitable cessation strategies into tobacco control efforts can support smoking cessation for everyone.

## Abstract

Tobacco dependence is a chronic condition driven by nicotine addiction. Successful quitting can be increased by health care provider intervention and evidence-based treatment. CDC assessed national estimates of cigarette smoking cessation indicators among U.S. adults using 2022 National Health Interview Survey data. In 2022, approximately two thirds (67.7%) of the 28.8 million U.S. adults who smoked wanted to quit, and approximately one half (53.3%) made a quit attempt, but only 8.8% quit smoking. One half of adults who smoked and saw a health professional during the past year received health professional advice (50.5%) or assistance (49.2%) to quit smoking. Among those who tried to quit, 38.3% used treatment (i.e., counseling or medication). Adults who usually smoked menthol (versus nonmenthol) cigarettes had higher prevalences of quitting interest (72.2% versus 65.4%; p<0.05) and past-year quit attempts (57.3% versus 50.4%; p<0.05), lower prevalences of receiving quit advice (48.2% versus 53.8%; p<0.05) and using cessation treatment (35.2% versus 41.5%; p<0.05), but similar prevalence of quit success (9.5% versus 7.9%; p = 0.19). Opportunities exist for both public health and health care sectors to increase smoking cessation, including expanding access to and utilization of cessation services and supports. Incorporating equitable cessation strategies into all commercial tobacco prevention and control efforts can help advance and support smoking cessation for all population groups.

## Introduction

Quitting smoking reduces the risk for premature death and smoking-related diseases (*1*). Tobacco dependence is a chronic, relapsing condition driven by nicotine addiction, and quitting can be difficult (*1*). Social and structural barriers to quitting exist differentially among population groups (*2*). For example, whereas comprehensive, barrier-free insurance coverage of cessation treatment is known to increase quitting success, only 20 state Medicaid programs provided such coverage in 2022 (*1*,*3*). In addition, commercial factors, such as marketing and product design, can influence quitting behaviors (*1*,*4*,*5*). Evidence suggests that persons who smoke menthol (versus nonmenthol) cigarettes could be less likely to successfully quit (*5*); previous studies have shown this finding is especially true of Black or African American (Black) adults who smoke, a high proportion of whom smoke menthol cigarettes in part because of aggressive, targeted marketing of menthol cigarettes to this population group (*2*,*5*).

Quitting success is increased by health care provider intervention and by use of behavioral counseling and Food and Drug Administration–approved medications, particularly when these treatments are used together (*1*). Understanding quitting intentions and behaviors can help identify gaps in treatment use and facilitate the development and implementation of efforts to increase access to and use of treatment. Healthy People 2030* includes four cessation-related objectives: 1) increasing quit attempts (TU-11), 2) increasing successful cessation (TU-14), 3) increasing receipt of health care provider advice to quit (TU-12), and 4) increasing treatment use (TU-13). This study expands on previous publications describing cessation-related indicators, including exploring differences in these indicators by sociodemographic and health-related factors as well as by cigarette type (menthol versus nonmenthol) (*1*).

## Methods

### Data Source

The National Health Interview Survey is an annual, nationally representative household survey of noninstitutionalized U.S. civilians. In 2022, a total of 27,651 adults aged ≥18 years were surveyed (response rate = 47.7%).^†^ Data were weighted to provide nationally representative estimates, adjusting for differences in selection probability and nonresponse. Consistent with previous studies, current smoking was defined as having ever smoked at least 100 cigarettes and currently smoking every day or some days (*1*). Former smoking was defined as having ever smoked at least 100 cigarettes and not currently smoking (*1*).

### Smoking Cessation Indicators

Seven smoking cessation indicators were assessed: 1) interest in quitting, 2) past-year quit attempt (trying to quit smoking or successfully quitting in the past year), 3) recent successful cessation (former smoking and quit for ≥6 months in the past year), 4) receipt of health professional advice to quit tobacco use, 5) receipt of health professional assistance to quit (advice about ways to quit or prescription of cessation medication),^§^ 6) use of counseling to quit,^¶^ and 7) use of medication to quit.**

### Data Analysis

Prevalence estimates were calculated for each cessation indicator overall and by sociodemographic and health characteristics. Differences in cessation indicators were assessed by usual type of cigarette smoked (menthol versus nonmenthol) both overall and among non-Hispanic White (White), non-Hispanic Black, and Hispanic or Latino (Hispanic) adults. Differences were assessed using Wald F chi-square tests with p-values <0.05 considered statistically significant. Analyses were conducted using SAS (version 9.4; SAS Institute) and SAS-callable SUDAAN (version 11.0.3; RTI International). This activity was reviewed by CDC, deemed not research, and conducted consistent with applicable federal law and CDC policy.^††^

## Results

### Cessation Indicators

In 2022, 11.6% (95% CI = 11.1%–12.1%; estimated 28.8 million) of U.S. adults reported current cigarette smoking. Approximately two thirds of adults (67.7%) wanted to quit smoking, and approximately one half (53.3%) tried to quit in the past year, but fewer than one in 10 (8.8%) recently successfully quit (Table 1).

**TABLE 1:** Prevalence of interest in quitting smoking,* past-year quit attempt,^†^ and recent successful smoking cessation^§^ among adults aged ≥18 years, by selected characteristics — National Health Interview Survey, United States, 2022

Characteristic	% (95% CI)		
	Interested in quitting	Past-year quit attempt	Recent successful cessation
**Overall**	**67.7 (65.7–69.7)**	**53.3 (51.4–55.1)**	**8.8 (7.7–9.9)**
**Sex**			
Men	67.1 (64.4–69.8)	53.4 (50.8–56.0)	8.7 (7.2–10.4)
Women	68.5 (65.5–71.5)	53.1 (50.1–56.0)	8.9 (7.5–10.5)
**Age group, yrs**			
18–24	56.5 (43.1–69.2)	74.4 (63.9–83.1)	15.3 (9.3–23.2)
25–44	70.2 (66.7–73.5)	57.9 (54.8–60.9)	12.4 (10.4–14.7)
45–64	69.9 (66.8–72.8)	47.5 (44.5–50.4)	5.6 (4.2–7.3)
≥65	60.1 (55.5–64.5)	48.6 (44.7–52.6)	5.6 (3.9–7.7)
**Race and ethnicity^¶^**			
AI/AN	—**	—**	—**
Asian	53.5 (39.5–67.1)	59.5 (47.1–71.0)	—**
Black or African American	70.3 (64.5–75.6)	57.1 (51.7–62.4)	7.3 (4.8–10.5)
White	68.4 (66.0–70.8)	50.9 (48.7–53.0)	8.7 (7.5–10.1)
Hispanic or Latino	64.2 (57.7–70.4)	56.0 (50.2–61.7)	10.9 (7.7–14.9)
Other	75.4 (60.1–87.1)	70.7 (58.2–81.3)	—**
**U.S. Census Bureau region^††^**			
Northeast	68.7 (63.4–73.6)	56.0 (50.8–61.2)	8.3 (5.8–11.4)
Midwest	68.9 (65.3–72.4)	51.1 (47.6–54.5)	8.2 (6.3–10.3)
South	67.4 (63.9–70.8)	53.0 (50.1–55.9)	8.4 (6.8–10.3)
West	66.1 (61.5–70.4)	54.5 (49.8–59.1)	10.9 (8.2–14.2)
**Urbanization level^§§^**			
Urban	68.4 (66.1–70.6)	54.1 (52.0–56.2)	9.6 (8.4–11.0)
Rural	65.4 (60.6–70.1)	50.0 (45.9–54.2)	5.5 (4.0–7.4)
**Educational attainment (among adults aged ≥25 yrs)**			
0–12 yrs, no diploma	64.3 (58.7–69.5)	47.7 (42.7–52.8)	4.0 (2.3–6.2)
GED	66.4 (57.1–74.9)	52.3 (44.0–60.4)	—**
High school diploma	69.2 (65.6–72.7)	51.3 (47.7–54.8)	7.4 (5.7–9.3)
Some college, no degree	69.0 (64.1–73.6)	52.5 (47.8–57.1)	9.4 (7.1–12.0)
Associate degree (academic, technical, or vocational)	73.6 (68.2–78.6)	51.5 (46.0–57.0)	8.3 (5.5–12.0)
Bachelor’s degree	68.4 (62.4–74.0)	55.8 (50.1–61.4)	14.8 (11.0–19.4)
Graduate degree (master’s, doctoral, or professional)	67.8 (57.9–76.6)	64.9 (55.6–73.5)	16.8 (10.6–24.6)
**Income to poverty ratio (income level)^¶¶^**			
0–1.99 (low)	65.6 (62.4–68.8)	52.9 (49.9–55.9)	7.5 (6.1–9.2)
2.00–3.99 (middle)	68.8 (65.0–72.5)	52.8 (49.2–56.4)	7.8 (6.2–9.7)
≥4.00 (high)	70.0 (66.0–73.7)	54.4 (50.6–58.1)	11.9 (9.5–14.6)
**Sexual orientation**			
Heterosexual or straight	68.0 (65.8–70.0)	52.2 (50.2–54.1)	8.1 (7.0–9.3)
Bisexual	61.2 (48.1–73.3)	65.8 (54.7–75.7)	23.7 (15.4–33.6)
Lesbian or gay	69.6 (53.6–82.7)	67.5 (54.3–78.1)	—**
Health insurance coverage***			
Private	70.9 (68.0–73.7)	54.7 (51.9–57.5)	10.0 (8.4–11.8)
Medicaid (including dual eligibility)	66.0 (61.5–70.3)	53.5 (49.6–57.4)	9.2 (7.0–11.8)
Medicare only (aged ≥65 yrs)	62.4 (55.1–69.4)	50.0 (43.2–56.8)	5.3 (3.0–8.7)
Other public insurance	64.5 (57.2–71.4)	52.8 (46.1–59.3)	8.2 (5.2–12.5)
Uninsured	64.3 (59.0–69.5)	49.5 (43.9–55.0)	6.2 (4.1–8.9)
**Disability^†††^**			
Yes	64.6 (59.2–69.8)	53.7 (48.8–58.7)	7.7 (5.3–10.9)
No	68.3 (66.0–70.4)	53.2 (51.1–55.2)	9.0 (7.8–10.2)
**Chronic disease diagnosis^§§§^**			
Smoking-related chronic disease	68.7 (65.0–72.3)	53.1 (49.6–56.6)	7.8 (5.9–10.2)
Other chronic disease	69.2 (66.1–72.2)	55.6 (52.6–58.5)	8.7 (7.0–10.7)
No chronic disease	65.2 (61.5–68.8)	51.2 (47.8–54.5)	9.6 (7.8–11.6)
**Anxiety disorder^¶¶¶^**			
Yes	71.2 (67.4–74.8)	59.5 (56.0–63.0)	9.7 (7.6–12.1)
No	66.4 (63.9–68.8)	50.8 (48.5–53.0)	8.4 (7.2–9.7)
Depression****			
Yes	69.4 (65.6–73.1)	58.8 (55.5–62.0)	9.3 (7.4–11.5)
No	67.0 (64.5–69.4)	50.9 (48.6–53.2)	8.6 (7.2–9.7)
**Mental health counseling (past year)^††††^**			
Yes	70.9 (66.0–75.4)	64.8 (60.1–69.3)	12.0 (9.2–15.2)
No	67.1 (64.8–69.4)	50.9 (48.9–53.0)	8.1 (7.1–9.4)

**Abbreviations:** AI/AN = American Indian or Alaska Native; GED = general educational development certificate; NCHS = National Center for Health Statistics.

* Adults who currently smoked cigarettes who reported that they wanted to stop smoking completely.

^†^ Adults who currently smoked cigarettes who reported that they stopped smoking for >1 day during the past 12 months because they were trying to quit smoking and adults who quit smoking during the past year among adults who currently smoke cigarettes and adults who quit smoking during the past year.

^§^ Adults who formerly smoked who quit smoking for ≥6 months during the past year among adults who currently smoke cigarettes (and have smoked for ≥2 years) and adults who quit smoking during the past year.

^¶^ Hispanic or Latino persons could be of any race. The group “Other, non-Hispanic” includes adults who were categorized as “non-Hispanic AI/AN and any other group” or “other single and multiple races” in the National Health Interview Survey public use file. All other groups were non-Hispanic, single-race categories.

** Estimates were statistically unreliable based on NCHS standards. https://www.cdc.gov/nchs/data/series/sr_02/sr02_175.pdf

^††^ https://www2.census.gov/geo/pdfs/maps-data/maps/reference/us_regdiv.pdf

^§§^ Based on the 2013 NCHS Urban-Rural Classification Scheme for Counties (https://www.cdc.gov/nchs/data/series/sr_02/sr02_166.pdf). For this report, the four-level urbanization level variable available in the public use data file is reduced to a dichotomous variable.

^¶¶^ Ratio of family income to poverty threshold for family size, based on the imputed family income to poverty threshold variable.

*** Private: any private insurance plan; Medicaid: Medicaid or other state-sponsored health plans, including the Children’s Health Insurance Program, or dual-enrolled in Medicare and Medicaid or other state-sponsored health plan; Medicare only: adults aged ≥65 years who had only Medicare coverage; other public insurance: any type of military coverage, coverage from other government programs, or Medicare among adults aged <65 years; and uninsured: no health insurance coverage. Insurance coverage is as of time of survey.

^†††^ Disability was defined based on self-reported presence of selected limitations, including vision, hearing, mobility, remembering or concentrating, self-care, and communication. Respondents who indicated “A lot of difficulty” or “Cannot do at all/unable to do” to questions related to these elements were coded as living with a disability; those who responded “no difficulty” or “some difficulty” were coded as having no disability. https://www.cdc.gov/nchs/washington_group/index.htm

^§§§^ Respondent ever told by a health professional that they had a specific chronic disease, including smoking-related chronic disease (cancer of the bladder; cervix; colon or rectum; esophagus; head and neck; larynx; lung; liver; mouth, tongue, or lip; pancreas; stomach; throat or pharynx; or uterus; chronic obstructive pulmonary disease, emphysema, or chronic bronchitis; angina; coronary heart disease; myocardial infarction; stroke; or type 2 diabetes); other chronic disease (cancer of the blood, bone, brain, breast, gallbladder, ovary, prostate, skin [melanoma or nonmelanoma], or thyroid; leukemia, lymphoma, melanoma, or other type of cancer; asthma; arthritis; chronic fatigue syndrome; dementia; epilepsy; high cholesterol; hypertension; or type 1 diabetes); or no chronic disease.

^¶¶¶^ Respondent ever told by a health professional that they had any type of anxiety disorder.

**** Respondent ever told by a health professional that they had any type of depression.

^††††^ Received counseling or therapy from a mental health professional such as a psychiatrist, psychologist, psychiatric nurse, or clinical social worker during the past year.

Among adults who currently smoked or who quit in the last year, 77.6% (95% CI = 75.7%–79.4%) and 83.1% (95% CI = 78.6%–87.1%), respectively, saw a health care provider in the past year. Among these adults, approximately one half received health professional advice (50.5%) or assistance (49.2%) to quit smoking (Table 2). Fewer than four in 10 (38.3%) adults who made a past-year quit attempt or quit smoking during the past 2 years used evidence-based treatment (counseling or medication) to help them quit. Medication^§§^ was used more commonly than counseling^¶¶^ (36.3% versus 7.3%). Very few used both medication and counseling (5.3%; 95% CI = 4.3%–6.4%).

**TABLE 2:** Prevalence of receiving health professional advice to quit smoking,* health professional assistance to quit smoking,^†^ and use of counseling^§^ and medication^¶^ for cessation among adults aged ≥18 years, by selected characteristics — National Health Interview Survey, United States, 2022

Characteristic	% (95% CI)				
	Received health professional advice to quit	Received health professional assistance to quit	Used counseling	Used medication	Used counseling or medication
**Overall**	**50.5 (48.4–52.6)**	**49.2 (47.0–51.4)**	**7.3 (6.1–8.6)**	**36.3 (33.9–38.6)**	**38.3 (36.0–40.6)**
**Sex**					
Men	47.7 (44.6–50.9)	46.4 (43.3–49.5)	6.9 (5.4–8.7)	34.7 (31.5–38.0)	36.9 (33.7–40.2)
Women	53.5 (50.6–56.3)	52.2 (49.3–55.1)	7.8 (6.1–9.8)	38.2 (34.9–41.7)	40.0 (36.6–43.4)
**Age group, yrs**					
18–24	32.5 (21.0–45.7)	31.0 (19.9–43.9)	—**	26.9 (18.1–37.3)	28.3 (19.4–38.7)
25–44	37.2 (33.7–40.9)	38.1 (34.6–41.6)	5.5 (3.9–7.6)	28.6 (25.2–32.2)	30.9 (27.4–34.5)
45–64	60.3 (56.9–63.6)	56.4 (52.9–59.9)	9.1 (7.1–11.5)	44.7 (40.8–48.7)	46.8 (42.8–50.7)
≥65	59.9 (55.6–64.1)	60.1 (55.9–64.3)	10.2 (7.2–13.8)	45.7 (40.3–51.1)	47.3 (42.0–52.6)
**Race and ethnicity^††^**					
AI/AN	—**	—**	—**	—**	—**
Asian	34.3 (21.5–49.0)	41.8 (29.9–55.6)	—**	—**	15.9 (7.7–27.8)
Black or African American	49.5 (43.9–55.1)	48.8 (42.5–55.2)	11.3 (8.1–15.3)	28.8 (23.6–34.5)	32.6 (27.2–38.2)
White	54.4 (51.9–57.0)	51.7 (49.0–54.4)	6.7 (5.3–8.4)	41.1 (38.2–44.1)	42.7 (39.8–45.7)
Hispanic or Latino	33.4 (27.0–40.2)	36.4 (30.3–42.8)	7.0 (4.1–11.1)	26.6 (20.8–33.0)	28.8 (22.9–35.3)
Other	45.7 (31.7–60.1)	42.6 (29.1–56.9)	—**	28.8 (17.3–42.8)	33.6 (22.1–48.1)
**U.S. Census Bureau region^§§^**					
Northeast	59.1 (53.7–64.4)	55.4 (49.5–61.1)	7.5 (4.5–11.4)	42.5 (36.9–48.2)	44.0 (38.3–49.8)
Midwest	54.9 (50.8–59.0)	52.1 (47.8–56.3)	8.4 (5.8–11.6)	38.3 (33.2–43.6)	40.1 (35.0–45.4)
South	46.2 (43.0–49.5)	46.4 (42.9–49.8)	5.9 (4.4–7.8)	33.7 (30.3–37.2)	35.5 (32.2–39.0)
West	46.7 (41.8–51.6)	46.4 (41.1–51.8)	9.0 (6.3–12.3)	34.3 (28.7–40.2)	37.4 (31.9–43.1)
**Urbanization level^¶¶^**					
Urban	49.6 (47.3–51.9)	48.7 (46.3–51.2)	7.7 (6.3–9.1)	35.8 (33.3–38.5)	38.0 (35.4–40.6)
Rural	53.9 (49.2–58.5)	51.1 (46.0–56.1)	5.7 (3.3–9.0)	38.1 (32.9–43.5)	39.6 (34.5–45.0)
**Educational attainment (among adults aged ≥25 yrs)**					
0–12 yrs, no diploma	51.5 (45.7–57.3)	52.7 (46.7–58.7)	5.6 (3.2–9.0)	31.2 (25.3–37.6)	32.6 (26.6–39.0)
GED	56.4 (46.4–66.0)	52.4 (42.4–62.3)	—**	40.7 (29.7–52.4)	43.6 (32.5–55.2)
High school diploma	51.3 (47.3–55.2)	50.0 (45.9–54.1)	4.4 (2.8–6.6)	33.3 (29.0–37.7)	34.6 (30.3–39.1)
Some college, no degree	49.0 (44.1–53.9)	49.2 (44.3–54.1)	11.5 (8.1–15.7)	41.0 (35.1–47.2)	44.1 (38.0–50.2)
Associate degree (academic, technical, or vocational)	56.9 (51.2–62.5)	51.3 (45.7–57.0)	12.0 (8.0–17.0)	39.1 (32.5–46.0)	43.2 (36.6–50.0)
Bachelor’s degree	46.7 (40.5–53.0)	45.7 (39.8–51.6)	7.0 (4.2–10.7)	43.9 (37.5–50.4)	45.3 (38.9–51.8)
Graduate degree (master’s, doctoral, or professional)	48.4 (38.4–58.5)	45.6 (36.1–55.4)	—**	40.1 (29.8–51.1)	40.8 (30.5–51.8)
Income to poverty ratio (income level)***					
0–1.99 (low)	52.1 (48.8–55.3)	51.8 (48.4–55.1)	8.4 (6.7–10.5)	33.6 (30.1–37.2)	36.0 (32.5–39.7)
2.00–3.99 (middle)	49.2 (45.3–53.1)	48.3 (44.3–52.3)	7.4 (5.4–9.9)	35.7 (31.5–40.0)	37.3 (33.1–41.7)
≥4.00 (high)	49.4 (45.7–53.7)	46.3 (42.2–50.3)	5.4 (3.6–7.8)	40.8 (36.2–45.5)	42.5 (37.9–47.3)
**Sexual orientation**					
Heterosexual or straight	50.9 (48.7–53.2)	49.5 (47.2–51.8)	7.0 (5.8–8.4)	36.0 (33.5–38.5)	37.9 (35.5–40.4)
Bisexual	48.1 (36.3–60.1)	46.7 (35.0–58.7)	—**	41.0 (29.4–53.4)	42.8 (31.1–55.0)
Lesbian or gay	41.2 (28.1–55.2)	56.8 (41.8–71.1)	—**	—**	—**
**Health insurance coverage^†††^**					
Private	48.5 (45.3–51.7)	46.5 (43.4–49.7)	5.9 (4.6–7.5)	37.9 (34.5–41.4)	39.2 (35.8–42.8)
Medicaid (includes dual eligibility)	55.6 (51.2–59.8)	56.0 (51.6–60.4)	9.5 (6.8–13.0)	39.5 (34.3–44.9)	42.5 (37.3–47.8)
Medicare only (aged ≥65 yrs)	62.8 (55.7–69.6)	61.1 (53.9–68.0)	8.9 (4.9–14.7)	43.0 (34.9–51.4)	43.4 (35.3–51.8)
Other public insurance	60.4 (53.0–67.6)	61.6 (54.6–68.2)	14.7 (8.9–22.4)	50.0 (41.1–59.0)	53.0 (44.1–61.8)
Uninsured	31.2 (23.7–39.4)	27.4 (20.4–35.4)	—**	17.2 (12.3–23.0)	20.4 (15.0–26.6)
**Disability^§§§^**					
Yes	63.7 (58.7–68.5)	63.3 (58.1–68.3)	10.5 (7.0–15.1)	47.1 (40.9–53.4)	49.8 (43.6–56.0)
No	47.9 (45.7–50.1)	46.4 (44.1–48.8)	6.7 (5.6–8.1)	34.4 (31.9–37.0)	36.3 (33.8–38.9)
**Chronic disease diagnosis^¶¶¶^**					
Smoking-related chronic disease	69.0 (65.7–72.1)	65.9 (62.6–69.1)	10.9 (8.3–13.9)	50.3 (46.0–54.5)	52.5 (48.3–56.7)
Other chronic disease	49.8 (46.5–53.0)	48.9 (45.3–52.4)	7.3 (5.4–9.4)	36.5 (32.9–40.2)	38.4 (34.7–42.2)
No chronic disease	32.1 (28.2–36.2)	32.2 (28.2–36.3)	4.7 (3.1–6.9)	25.8 (21.9–30.1)	27.8 (23.8–32.1)
Anxiety disorder****					
Yes	56.6 (52.8–60.4)	54.6 (50.6–58.4)	12.3 (9.6–15.5)	44.9 (40.5–49.3)	48.3 (44.0–52.6)
No	46.7 (45.2–50.3)	46.8 (44.1–49.5)	4.9 (3.9–6.2)	32.2 (29.4–35.2)	33.6 (30.7–36.6)
**Depression^††††^**					
Yes	55.8 (52.1–59.4)	54.5 (50.9–58.1)	11.7 (9.2–14.7)	47.8 (43.6–52.1)	50.5 (46.3–54.7)
No	47.8 (45.1–50.5)	46.7 (43.9–49.4)	5.1 (3.9–6.4)	30.5 (27.7–33.4)	32.2 (29.4–35.1)
**Mental health counseling (past year)^§§§§^**					
Yes	51.9 (47.4–56.5)	54.7 (50.0–59.3)	15.1 (11.4–19.4)	45.6 (40.3–51.0)	49.1 (43.8–54.4)
No	50.1 (47.7–52.5)	47.7 (45.2–50.2)	5.2 (4.2–6.4)	33.8 (31.2–36.5)	35.4 (32.8–38.1)

**Abbreviations:** AI/AN = American Indian or Alaska Native; GED = general educational development certificate; NCHS = National Center for Health Statistics.

* Received advice from a health professional to quit tobacco use. Prevalence was measured among respondents who currently smoked cigarettes and respondents who quit smoking during the past 12 months who had seen a doctor, other health professional, or mental health professional during the past year.

^†^ Received assistance from a health professional to quit (i.e., advice about ways to quit smoking or prescription of cessation medication). Prevalence was measured among respondents who currently smoked cigarettes and respondents who quit smoking during the past 12 months who had seen a doctor, other health professional, or mental health professional during the past year.

^§^ Used one-on-one counseling; a stop smoking clinic, class, or support group; a telephone help line or quitline; or more than one of these modalities to stop smoking. Prevalence was measured among respondents who currently smoked who tried to quit during the past year and respondents who quit smoking during the past 2 years.

^¶^ Used nicotine patch, nicotine gum or lozenge, nicotine nasal spray or inhaler, varenicline, bupropion, or more than one of these medications to stop smoking. Prevalence was measured among respondents who currently smoked who tried to quit during the past year and respondents who quit smoking during the past 2 years.

** Estimates were statistically unreliable based on NCHS standards. https://www.cdc.gov/nchs/data/series/sr_02/sr02_175.pdf

^††^ Hispanic or Latino persons could be of any race. The group “Other, non-Hispanic” includes adults who were categorized as “non-Hispanic AI/AN and any other group” or “other single and multiple races” in the National Health Interview Survey public use file. All other groups were non-Hispanic, single-race categories.

^§§^ https://www2.census.gov/geo/pdfs/maps-data/maps/reference/us_regdiv.pdf

^¶¶^ Based on the 2013 NCHS Urban-Rural Classification Scheme for Counties (https://www.cdc.gov/nchs/data/series/sr_02/sr02_166.pdf). For this report, the four-level urbanization level variable available in the public use data file is reduced to a dichotomous variable.

*** Ratio of family income to poverty threshold for family size, based on the imputed family income to poverty threshold variable.

^†††^ Private: any private insurance plan; Medicaid: Medicaid or other state-sponsored health plans, including the Children’s Health Insurance Program, or dual-enrolled in Medicare and Medicaid or other state-sponsored health plan; Medicare only: adults aged ≥65 years who had only Medicare coverage; other public insurance: any type of military coverage, coverage from other government programs, or Medicare among adults aged <65 years; and uninsured: no health insurance coverage. Insurance coverage is as of time of survey.

^§§§^ Disability was defined based on self-reported presence of selected limitations, including vision, hearing, mobility, remembering or concentrating, self-care, and communication. Respondents who indicated “A lot of difficulty” or “Cannot do at all/unable to do” to questions related to these elements were coded as living with a disability; those who responded “no difficulty” or “some difficulty” were coded as having no disability. https://www.cdc.gov/nchs/washington_group/index.htm

^¶¶¶^ Respondent ever told by a health professional that they had a specific chronic disease, including smoking-related chronic disease (cancer of the bladder; cervix; colon or rectum; esophagus; head and neck; larynx; lung; liver; mouth, tongue, or lip; pancreas; stomach; throat or pharynx; or uterus; chronic obstructive pulmonary disease, emphysema, or chronic bronchitis; angina; coronary heart disease; myocardial infarction; stroke; or type 2 diabetes); other chronic disease (cancer of the blood, bone, brain, breast, gallbladder, ovary, prostate, skin [melanoma or nonmelanoma], or thyroid; leukemia, lymphoma, melanoma, or other type of cancer; asthma; arthritis; chronic fatigue syndrome; dementia; epilepsy; high cholesterol; hypertension; or type 1 diabetes); or no chronic disease.

**** Respondent ever told by a health professional that they had any type of anxiety disorder.

^††††^ Respondent ever told by a health professional that they had any type of depression.

^§§§§^ Received counseling or therapy from a mental health professional such as a psychiatrist, psychologist, psychiatric nurse, or clinical social worker during the past year.

### Cessation Indicators by Sociodemographic and Health Characteristics

Cessation indicators varied by sociodemographic and health characteristics. For example, prevalence of past-year quit attempts ranged from 74.4% among persons aged 18–24 years to 47.5% among those aged 45–64 years (Table 1). Recent successful quitting ranged from 15.3% among those aged 18–24 years to 5.6% among those aged 45–64 and ≥65 years. Recent successful quitting also varied by education (ranging from 16.8% among those with a graduate degree to 4.0% among those without a high school diploma) and income level (ranging from 11.9% among those with high income to 7.5% among those with low income). Treatment use varied by race and ethnicity. Prevalence was 42.7% among White adults, followed by non-Hispanic adults of another race (33.6%), Black adults (32.6%), Hispanic adults (28.8%), and non-Hispanic Asian adults (15.9%) (Table 2). When stratified by insurance coverage, prevalences of receiving advice, receiving assistance, and using any treatment were lowest among uninsured adults (31.2%, 27.4%, and 20.4%, respectively). Adults reporting a smoking-related chronic disease, anxiety disorder, depression, or disability had higher prevalences of receiving advice or assistance and of using treatment than did adults without these conditions.

### Cessation Indicators by Cigarette Type Smoked (Menthol Versus Nonmenthol)

Adults who usually smoked menthol (versus nonmenthol) cigarettes had higher prevalences of interest in quitting (72.2% versus 65.4%; p<0.05) and quit attempts (57.3% versus 50.4%; p<0.05), but a similar prevalence of recent successful cessation (9.5% versus 7.9%; p = 0.19) (Figure). Adults who smoked menthol (versus nonmenthol) cigarettes had lower prevalences of receiving advice to quit (48.2% versus 53.8%; p<0.05) and using treatment (35.2% versus 41.5%; p<0.05).

**FIGURE:**
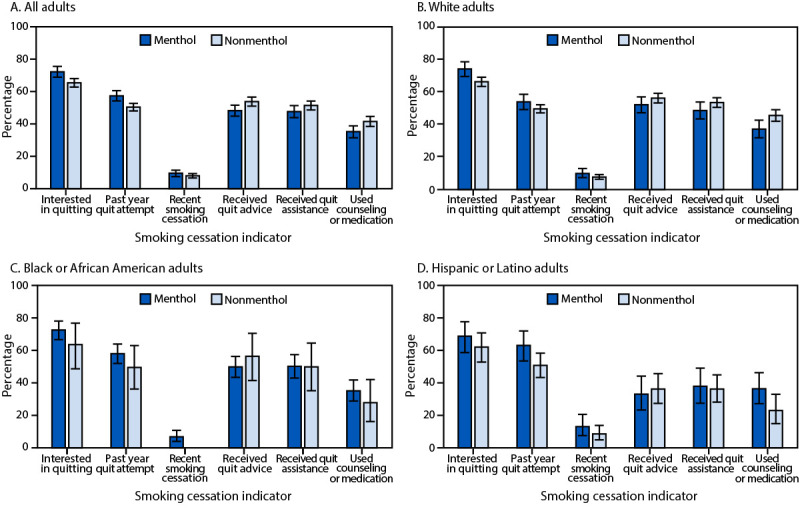
Prevalence* of interest in quitting smoking,^†^ past-year quit attempt,^§^ recent successful smoking cessation,^¶^ receiving health professional advice to quit,** receiving health professional assistance to quit,^††^ and use of counseling or medication^§§^ for cessation among adults aged ≥18 years, by race and ethnicity^¶¶^ and type of cigarette usually smoked***^,^^†††^ — National Health Interview Survey, United States, 2022 * With 95% CIs indicated by error bars. ^†^ Adults who currently smoked cigarettes who reported wanting to stop smoking completely. ^§^ Adults who currently smoked cigarettes who reported that they stopped smoking for >1 day during the past 12 months because they were trying to quit smoking and adults who quit smoking during the past year among adults who currently smoke cigarettes and adults who quit smoking during the past year. ^¶^ Adults who formerly smoked and quit smoking for ≥6 months during the past year among adults who currently smoke cigarettes (and have smoked for ≥2 years) and adults who quit smoking during the past year. ** Received advice from a health professional to quit tobacco use. Prevalence was measured among respondents who currently smoked cigarettes and respondents who quit smoking during the past 12 months who had seen a doctor, other health professional, or mental health professional during the past year. ^††^ Received assistance from a health professional to quit (i.e., advice about ways to quit smoking or prescription of cessation medication). Prevalence was measured among respondents who currently smoked cigarettes and respondents who quit smoking during the past 12 months who had seen a doctor, other health professional, or mental health professional during the past year. ^§§^ Used counseling (e.g., one-on-one counseling; a stop smoking clinic, class, or support group; a telephone help line or quitline; or more than one of these modalities) or medication (e.g., nicotine patch, nicotine gum or lozenge, nicotine nasal spray or inhaler, varenicline, bupropion, or more than one of these medications) to stop smoking. Prevalence was measured among respondents who currently smoked who tried to quit during the past year and respondents who quit smoking during the past 2 years. ^¶¶^ Hispanic or Latino (Hispanic) persons could be of any race. All other groups were non-Hispanic, single-race categories. *** Type of cigarette usually smoked reported as menthol or nonmenthol. Persons who reported no usual type were excluded from the analysis. ^†††^ The following differences were statistically significant (Wald F chi-square; p<0.05): all adults (interested in quitting, past-year quit attempt, received quit advice, and used counseling or medication); non-Hispanic White adults (interested in quitting and used counseling or medication); Hispanic adults (past-year quit attempt and used counseling or medication). The estimate for recent smoking cessation among non-Hispanic Black or African American adults who usually smoked nonmenthol cigarettes was statistically unreliable based on standards from the National Center for Health Statistics. https://www.cdc.gov/nchs/data/series/sr_02/sr02_175.pdf

## Discussion

In 2022, most adults who smoked wanted to quit, and approximately one half tried to quit in the past year, but fewer than 10% quit successfully. Consistent with previous studies, this analysis identified a low prevalence of clinical cessation intervention (i.e., advice and assistance) and treatment use (*1*). Several barriers to treatment access might play a part in this finding. Medication recalls and shortages have contributed to declines in prescriptions for cessation medication (*6*). Gaps exist in both clinician knowledge of cessation treatment and availability of comprehensive cessation-related clinical practice guidelines (*7*,*8*). Provision of cessation treatment in behavioral health settings and hospital-affiliated cessation programs is limited (*9*,*10*). Access barriers to Medicaid treatment coverage, such as treatment duration limits, annual limits on the number of covered quit attempts, and requirements for prior authorization, are common (*3*). In addition, threats to maintenance of current access exist, including recent discontinuation of the nicotine oral inhaler*** as well as pending legal challenges to requirements in the Affordable Care Act for most private insurers to cover tobacco cessation treatments.^†††^

The U.S. Department of Health and Human Services has identified opportunities to advance and support smoking cessation, including among population groups experiencing smoking- and cessation-related disparities.^§§§^ Comprehensive commercial tobacco^¶¶¶^ prevention and control strategies, such as retail strategies and smoke-free policies, can support and increase cessation at the population level (*1*). Equitable implementation of such strategies needs to include attention to ensuring equitable access to cessation treatments and supports. Leveraging and expanding the current infrastructure of evidence-based cessation supports, including quitlines,**** digital cessation services,^††††^ and cessation-focused mass media campaigns can continue to advance smoking cessation (*1*). Expanding and promoting barrier-free, comprehensive cessation treatment coverage can increase availability and use of treatment (*1*). In addition, implementing systems-level changes in health care settings, including adoption of treatment protocols and standardized clinical workflows, can systematize clinical treatment delivery and might increase treatment access for the approximately three in four adults who smoke who see a health care provider in a given year^§§§§^ (*1*). This analysis identified a lower prevalence of receiving clinician advice and assistance to quit smoking among adults without smoking-related disease. Systemization of treatment delivery could help ensure clinical intervention for all adults who smoke, thereby potentially increasing the prevention of smoking-related disease and death (*1*). 

As efforts toward advancing cessation continue, awareness of tobacco-related disparities and attention to the unique needs of each population group (e.g., cultural and language preferences and treatment access barriers) remain critical to ensuring equitable progress. For example, in this study, adults who smoked menthol (versus nonmenthol) cigarettes had a similarly low prevalence of quit success despite higher prevalences of quitting interest and quit attempts. This finding might be due, in part, to lower use of treatment in this group, which suggests a need to enhance treatment awareness, access, and use among adults who smoke menthol cigarettes, particularly as jurisdictions enact restrictions on the sale of flavored tobacco products.^¶¶¶¶^ Substantial evidence shows that adoption of policies that prohibit the sale of menthol cigarettes increases smoking cessation and could help reduce tobacco-related health disparities (*5*). Increasing and ensuring equitable awareness of and access to cessation services, including counseling and medication, (i.e., taking a cessation in all tobacco policies approach) is important to maximizing the impact of commercial tobacco control policies, including flavor prohibitions.

### Limitations

The findings in this report are subject to at least two limitations. First, because the National Health Interview Survey does not sample institutionalized adults or adults in the military, results are not generalizable to these groups. Second, survey responses were self-reported and not biochemically validated and might be subject to social desirability and recall bias.

### Implications for Public Health Practice

Substantial progress has been made in reducing cigarette smoking in the United States, but disparities in use and cessation remain (*1*). Continued progress in reducing tobacco use and related disparities requires efforts to increase smoking cessation. Opportunities exist across public health and health care sectors to increase smoking cessation, including expanding access to and use of cessation services and supports. Incorporating equitable cessation opportunities into all commercial tobacco prevention and control efforts (i.e., taking a cessation in all tobacco policies approach) can help advance and support smoking cessation for all population groups and has potential to reduce tobacco-related health disparities.

## Footnotes

* https://health.gov/healthypeople/objectives-and-data/browse-objectives/tobacco-use

†
https://ftp.cdc.gov/pub/Health_Statistics/NCHS/Dataset_Documentation/NHIS/2022/srvydesc-508.pdf

§ Health professional advice and assistance were measured among respondents who currently smoked and respondents who quit smoking during the past 12 months who had seen a doctor, other health professional, or mental health professional during the past year.

¶ Used one-on-one counseling; a stop smoking clinic, class, or support group; a telephone help line or quitline; or more than one of these modalities to stop smoking. Prevalence was measured among respondents who currently smoked who tried to quit during the past year and respondents who quit smoking during the past 2 years.

** Used nicotine patch, nicotine gum or lozenge, nicotine nasal spray or inhaler, varenicline, bupropion, or more than one of these medications to stop smoking. Prevalence was measured among respondents who currently smoked who tried to quit during the past year and respondents who quit smoking during the past 2 years.

†† 45 C.F.R. part 46.102(l)(2), 21 C.F.R. part 56; 42 U.S.C. Sect. 241(d); 5 U.S.C. Sect. 552a; 44 U.S.C. Sect. 3501 et seq.

§§ The most commonly used medication was nicotine patch (19.6%), followed by nicotine gum or lozenge (18.4%), varenicline (9.6%), bupropion (6.4%), and nicotine spray or inhaler (1.0%).

¶¶ The most commonly used counseling modality was one-on-one counseling (4.3%), followed by telephone quitline (3.7%), and class, support group, or clinic (2.4%).

***
https://cdn.pfizer.com/pfizercom/DHCP_Letter_Nicotrol_Inhaler_5.30.2023.pdf

††† The Affordable Care Act requires most private insurance plans to cover treatment given an “A” or “B” grade by the U.S. Preventive Services Task Force (https://www.congress.gov/111/plaws/publ148/PLAW-111publ148.pdf). Tobacco cessation treatment currently receives an “A” grade (https://www.uspreventiveservicestaskforce.org/uspstf/recommendation/tobacco-use-in-adults-and-pregnant-women-counseling-and-interventions). This requirement is currently being challenged in the courts. https://www.kff.org/womens-health-policy/issue-brief/explaining-litigation-challenging-the-acas-preventive-services-requirements-braidwood-management-inc-v-becerra/

§§§ The U.S. Department of Health and Human Services Framework to Support and Accelerate Smoking Cessation outlines six goals, with supporting strategic opportunities, to advance smoking cessation in the United States. https://www.hhs.gov/sites/default/files/hhs-framework-support-accelerate-smoking-cessation-2024.pdf

¶¶¶ Commercial tobacco refers to tobacco products that are made and sold by companies. This definition does not include traditional tobacco used by some Indigenous groups for religious or ceremonial purposes.

**** All 50 states, the District of Columbia, Guam, and Puerto Rico have a tobacco cessation quitline with services free to callers, available through the national quitline portal (1-800-QUIT-NOW).

†††† Digital tobacco cessation services are available throughout the United States, including web-based interventions (www.smokefree.gov), and text-based interventions such as those available through the National Texting Portal (by texting QUITNOW to 333888). https://www.cdc.gov/tobacco/campaign/tips/quit-smoking/national-texting-portal.html

§§§§ The Million Hearts Tobacco Cessation Change Package outlines evidence-based and promising practice health systems changes to enhance and improve integration of tobacco dependence treatment into routine clinical care. https://millionhearts.hhs.gov/files/tobacco_cessation_change_pkg.pdf

¶¶¶¶
https://assets.tobaccofreekids.org/factsheets/0398.pdf
